# The Late Martin Gay, M. D.

**Published:** 1850-02

**Authors:** 


*The late Martin Gay, M. D.* Dr. Gay died of acute peritonitis. The history of his illness is interesting, in view of its relation to one so universally esteemed, and from the absence of diagnostic symptoms.

On Saturday, January 5th, he was, as usual, in good health. During the evening felt sick, and took some calomel and blue pill. On going to bed, had a severe rigor, which lasted an hour, notwithstanding the assiduous application of hot flannels, &c. He took some ipecac, and, during the night, vomited several times.

On Sunday morning he was visited by Dr. J. B. S. Jackson. He complained of distress in the head, and general soreness of the body. He had nausea, and was desirous of taking another emetic—having the feeling that the stomach and bowels needed to be relieved of their contents.

Dr. Jacksen advised him to resist the vomiting as far as possible, and to take a cathartic of senna. In the afternoon, by the aid of an enema, he had free dejections, and also vomited bilious fluid. For the next two days considered himself improving, and Dr. Jackson not being able to see him, he was not visited till Wednesday, P. M., when he sent for me.

He was in bed. Skin dry and warm, respiration natural. Pulse 70, quiet. Tongue moist—somewhat coated. He had eaten a little toast at breakfast, with great relish. No thirst; but in the course of the day he had drank large quantities of water, hoping thereby to induce perspiration. Bowels free—urine sufficient—no dysuria. General soreness on pressure of the whole body, but no one spot particularly tender. The rigor, nausea, and distress in head, with which the disease commenced, had recurred at intervals during each day; but the rigor was slight and momentary. The nausea was attended with a feeling of uneasiness and exhaustion, referred by him to the cardiac orifice. There was occasional vomiting of thin bilious fluid, with temporary relief. He had no positive pain in the head, but a sense of depressing, nauseating distress. Indeed, the chief complaint made was of exhaustion.

The nature of the disease was not apparent. He had a mild febrile affection, but the access and consequent series of symptoms were not those of typhoid fever. There was no pain in any internal part, and, with exception of nausea, no striking derangement of function. Nevertheless, after so formidable a rigor, one could not but suspect the existence of grave local inflammation. The absence of acute pain, together with the unembarrassed state of the cerebral functions, excluded the idea of meningitis. The abdomen was neither full nor tense. No tenderness on pressure at any point, though repeatedly and carefully examined. The bowels responded readily to cathartics, and the only signs of functional embarrassment were the sensation of nausea and of desire for further evacuation.

His own theory was, that the symptoms were owing to a "bilious disorder of the stomach," of which he had suffered two or three attacks, and which had been attended with rigor. He had been exposed to varioloid about a fortnight before his attack, but there were no pustules to be found, and he had not the characteristic violent pain in head and back.

In the absence, therefore, of any positive indication, I advised the sparing use of effervescing drink, a mustard foot bath, and sinapisms alternately to epigastrium and back of neck.

The next morning (Thursday) I found him sitting at his table in good spirits; he had slept in the night, and all his symptoms were mitigated. Pulse 80. Had taken some arrowroot with relish. During the day, however, nausea and vomiting returned, together with the feeling of sinking, referred to the cardiac orifice. He again proposed an emetic, but I dissuaded him, and advised a repetition of the baths, &c., and at bed time six grains each of calomel and compound ext. of colocynth. About 4 A. M. on Friday, had vomiting and three dejections, with a sense of very great relief; but within an hour was suddenly seized with extreme pain in middle of the sternum, extending thence to the epigastrium. I was called at 7. His countenance was pinched and haggard—mind perfectly clear. Pulse 120, retaining some force—distressing nausea and hiccup. He had taken paregoric and half a grain of opium. I directed morphia and the inhalation of ether. Ten leeches were applied to the epigastrium. During the day the pain was unabated and intolerable, unless when under the influence of ether, to which he resorted very frequently, and with the happiest effect. Pulse from 150 to 180. With the exception of a few short intervals, his mind was clear until within half an hour of his death, which occurred on Saturday afternoon.

On examination, after death, there was found universal inflammation of the peritoneal surface of the intestines and of the stomach. The convolutions united slightly with delicate recent lymph. There was effusion of a few ounces of serum, and, in the cavity of the pelvis, a little purulent fluid. The mucous surface of the stomach healthy—considerable injection of the vessels at the cardiac orifice.

The most remarkable feature in this case was the absence of pain and other diagnostic marks of peritonitis. I might have distrusted my examination had it not been confirmed by other medical gentlemen who visited him. Latent peritonitis, though rare, has been fully exemplified. I can refer to six cases that have occurred in this city within the last ten years, under the care of different gentlemen well known in the profession.

Another peculiarity was the temporary amendment, suddenly followed by violent and fatal increase of disease. It would seem that, after the first onset, the inflammation—comparatively dormant—was confined to a small space, and then suddenly excited, it spread from below upwards over the whole abdomen.

C. G. PUTNAM.

*Boston, Jan* 21, 1850. [*Bost. Med. and Surg. Jour.*

---

*Report to the Erie (Pennsylvania) County Medical Society, on the prevalence of Suits for Mal-Practice.*

We find the following report in the columns of the Erie (Penn.) Gazette. The Chairman of the Committee, Dr. Wood, Surgeon U. S. Navy, is the author of an interesting paper on the same subject, communicated for the American Journal of Medical Sciences, and contained in Eclectic Department of the January aumber of this Journal. The subject is an important one, more especially to all who practice surgery, and we suggest that, with a view to excite attention to it, and to give a proper direction to the reflections of the public, it would be well for members of the profession in different places, to procure the insertion of this report in periodicals having

